# A Phase 1 Safety Study of Evexomostat (SDX-7320) in Patients with Late-Stage Cancer: An Antiangiogenic, Insulin-Sensitizing Drug Conjugate Targeting METAP2

**DOI:** 10.1158/2767-9764.CRC-24-0627

**Published:** 2025-06-23

**Authors:** Monica M. Mita, Alain C. Mita, Bradley J. Carver, James M. Shanahan, Benjamin A. Mayes, Pierre J. Dufour, David Browning, Alfred Anderson-Villaluz, John S. Petersen, David J. Turnquist, Peter Cornelius

**Affiliations:** 1 Hoag, Newport Beach, California.; 2 SynDevRx Inc., Cambridge, Massachusetts.; 3 Nuvation Bio, San Francisco, California.; 4 Glyscend Therapeutics, Lowell, Massachusetts.; 5 Hemavant Sciences, New York, New York.

## Abstract

**Purpose:**

To investigate the safety and tolerability of evexomostat (SDX-7320) in patients with late-stage cancer.

**Patients and Methods:**

This phase 1 dose-escalation safety study used an accelerated titration followed by a 3 + 3 design on 7- or 14-day administration, with dose expansion at the recommended phase 2 dose in 32 patients with late-stage, solid tumors. Measurements included standard assessments of safety, tolerability, target engagement in whole blood, plasma levels of protein biomarkers, and drug exposure. Tumor response was measured using RECIST v.1.1.

**Results:**

Thirty-two patients were dosed with evexomostat (SDX-7320), starting at 1.7 mg/m^2^ once per week and escalated to 65 mg/m^2^ (once every 2 weeks, 28 days/cycle). Dose escalation and expansion confirmed the maximum tolerated dose at 49 mg/m^2^ once every 2 weeks with reversible thrombocytopenia as the dose-limiting toxicity. Most treatment-emergent adverse events were of grade 1 or 2 in severity and nonserious, with no grade 5 adverse events. Eighty percent of patients (*n* = 20/25 evaluable) had stable disease, and the average treatment duration was 87 days (3.1 cycles). Key angiogenic biomarkers VEGF-C and bFGF (FGF2) improved in response to evexomostat. Patients with baseline insulin resistance (i.e., fasting insulin >20 µU/mL; *n* = 11) exhibited significant decreased fasting insulin after treatment. Decreases in leptin were observed in 27/31 patients (87%), whereas adiponectin increased in 28/31 patients (90%). Plasma lipid profiles showed increased high-density lipoprotein (HDL) and decreased low-density lipoprotein (LDL) cholesterol.

**Conclusions:**

Evexomostat (SDX-7320) was well-tolerated with prolonged stable disease and metastatic control in an open-label, phase I safety study. Improvements were observed in angiogenic and metabolic biomarkers.

**Significance:**

Obesity and insulin resistance are known to promote tumor growth and accelerate the mortality of patients with cancer. Evexomostat is a novel antiangiogenic and antimetastatic drug candidate which also has insulin-sensitizing and antiobesity properties that is being developed for use in combination with standard-of-care therapies for obese patients with cancer.

## Introduction

Methionine aminopeptidase-2 (METAP2) inhibitors have demonstrated potent antiangiogenic and antitumor effects in patients with late-stage cancer (1–3) as well as highly favorable metabolic effects, including significant weight loss and improvements in insulin sensitivity and blood lipid levels in obese patients (4, 5). Preclinically, METAP2 inhibition impacts multiple cellular signaling pathways and processes, including cell-cycle arrest via p21 induction and CDK2 inhibition (6), inhibition of noncanonical WNT signaling (7), inhibition of vascular mimicry (8), global systemic improvements in dysregulated metabolic hormones such as insulin, leptin, and adiponectin (4, 9–11), and reductions in fibrosis (12, 13). However, despite compelling clinical and preclinical data, no METAP2 inhibitor has been approved for clinical use due to toxicities and poor drug-like properties.

Fumagillin, a natural product isolated from the fungus *Aspergillus fumigatus Fresenius* (2), along with various fumagillin derivatives potently and irreversibly binds to METAP2 (14–16), inactivating its aminopeptidase activity, leading to the observed cascade of downstream biologic effects. Whereas the clinical benefits of METAP2 inhibition in patients with cancer and obese patients are well documented, small-molecule fumagillin-based METAP2 inhibitors can cause reversible central nervous system (CNS) toxicities and poor drug-like properties, necessitating frequent dosing (17, 18), thereby impeding their clinical development.

Evexomostat (SDX-7320) is a polymer–drug conjugate of a novel fumagillin-derived METAP2 inhibitor which mitigates CNS toxicity (relative to prior small-molecule METAP2 inhibitors) and is highly water-soluble, enabling its administration by subcutaneous injection (19). Evexomostat is intended for the treatment of solid tumors and, in particular, those sensitive to dysregulated metabolic hormones—an emerging area of oncology research referred to as metabo-oncology (19)*.* Metabolic dysfunction, a downstream sequela of obesity and/or hyperadiposity, enhances the growth and metastatic potential of certain tumor types (20). In this setting, the metabolic hormones leptin, adiponectin, and insulin have been implicated as agonists of key tumor signaling pathways leading to cancer progression and patient mortality (21–24). Evexomostat combines direct antimetastatic and antiangiogenic activities with global improvements in dysregulated metabolic hormones. Evexomostat was designed to prevent CNS-related toxicities associated with prior-generation small-molecule METAP2 inhibitors and improve drug-like properties (19). Evexomostat is being developed for combination treatment regimens in which antiangiogenic, antimetastatic, and metabolic-correcting effects may potentiate the efficacy of concomitant standard-of-care therapies. In this study, we report on the safety, maximum tolerated dose (MTD), recommended phase 2 dose (RP2D) and schedule of evexomostat monotherapy, as well as changes in angiogenic and metabolic biomarkers from a phase 1 first-in-human safety study in patients with heavily pretreated, late-stage cancer with a variety of solid tumors.

## Materials and Methods

### Study design

This phase 1, open-label, accelerated dose-escalation study (NCT02743637) was primarily intended to assess and characterize the safety and tolerability of evexomostat (SDX-7320) in patients with advanced refractory or late-stage solid tumors that had progressed through all standard treatment options, establish the single-agent MTD, and determine the RP2D and schedule for future clinical trials in combination with standard-of-care therapies. Evexomostat was administered subcutaneously on two dosing schedules: once weekly (Q7D) or once every 2 weeks (Q14D) in cycles of 28 days. Patients were initially enrolled into the Q7D dosing schedule. The study began with an accelerated, single-patient dose-escalation titration schema followed by a traditional 3 + 3 dose-escalation design once a grade 2 or greater toxicity was observed. Dose-escalation was based on a modified Fibonacci sequence schema. After the evexomostat MTD was determined on the Q7D schedule, enrollment on the Q14D dosing schedule commenced and continued until the MTD was determined. Up to six additional patients were treated at the MTD to further characterize the safety, pharmacokinetic (PK), and pharmacodynamic (PD) profile of the drug. Patients were treated with evexomostat until progression of their disease, unacceptable toxicity, or withdrawal of consent. Predose safety clinical and laboratory evaluations were conducted before the beginning of each cycle.

The NCI Common Terminology Criteria for Adverse Events, v4.03, was used to determine toxicity levels. An isolated laboratory finding must have been clinically significant according to the discretion of the investigator to be considered a dose-limiting toxicity (DLT). All safety data were monitored on an ongoing basis. A DLT was defined as any of the following adverse events (AE) that were clinically significant, occurred during cycle 1, persisted despite maximal medical support, and were deemed by the investigator to be possibly, probably, or definitely related to the administration of evexomostat: (i) any ≥ grade 3 nonhematologic toxicity lasting for 7 days; (ii) any ≥ grade 3 nausea, diarrhea, and/or vomiting lasting for 3 days, provided patient received maximal medical intervention and/or prophylactic antiemetic therapy; (iii) any ≥ grade 3 hematologic toxicity lasting 3 days; or (iv) any ≥ grade 3 febrile neutropenia.

The study was conducted at three centers in the United States: Cedars Sinai Medical Center, Los Angeles, CA; Sarah Cannon Research Institute, Nashville, TN; and HonorHealth, Scottsdale, AZ. The Institutional Review Board of each participating institution approved all study protocols and amendments. Written informed consent was obtained from all participants before any study specific procedures were performed. The study was conducted in accordance with the Declaration of Helsinki and International Council for Harmonisation (ICH) Good Clinical Practice guidelines.

Eligible patients were of ages between 21 and 85 years with histologically or cytologically confirmed advanced refractory or late-stage solid tumor who had progressed on standard therapy or for whom no effective anticancer therapy was available. Inclusion criteria included at least one site of radiographically measurable disease, Eastern Cooperative Oncology Group performance status ≤1, adequate renal and liver functions, and life expectancy ≥3 months. Patients with organ transplant surgery, a known history of hepatitis A, B, or C and on active antiviral therapy, history of gastric bypass surgery or banding procedure, uncontrolled or refractory hypertension (systolic >180 mm Hg or diastolic >110 mm Hg) or hypotension (systolic <90 mm Hg or diastolic <50 mm Hg) despite medical treatment, an electrocardiogram QTc (Fridericia correction) ≥470 ms, or a congenital prolonged QT syndrome were excluded from this study. Patients with a known primary brain malignancy or brain metastases, those requiring insulin for control of diabetes, or participants in a trial of another investigational agent within 30 days prior to first dose of study drug were also excluded. Representativeness of study participants is summarized in Supplementary Table S1.

### Evexomostat (SDX-7320) administration

Evexomostat formulation for injection was a sterile solution of evexomostat dissolved in aqueous D-mannitol (5% w/v). The investigational product was prepared by an approved pharmacist at a registered compounding pharmacy according to USP <797>. The evexomostat concentration (range, 2–20 mg/mL) was calculated as a function of the dose level and patient body surface area as appropriate for midabdominal subcutaneous injection.

### Doses and schedules

The starting dose of evexomostat was 1.7 mg/m^2^, and after 10 single-patient cohorts, reached a maximum of 49 mg/m^2^ with a total of 15 patients on the Q7D dosing schedule (administered subcutaneously on days 1, 8, 15, and 22, every 28 days). Seventeen patients were assigned to the Q14D cohort (administered subcutaneously on days 1 and 15, every 28 days); six patients at 36 mg/m^2^, six at 49 mg/m^2^, and five at 65 mg/m^2^. For the purposes of data analysis, the 10 single-patient Q7D cohorts assigned to evexomostat in the accelerated titration portion (doses of 1.7–36 mg/m^2^ Q7D, 2@36 mg/m^2^) were combined into one cohort.

### Study objectives and assessments

The primary study objectives were to determine the safety and tolerability of evexomostat and its MTD in unselected patients with advanced refractory solid tumors and determine the RP2D and dosing schedule for future studies. The secondary objectives were to evaluate the PK profile of the polymer–drug conjugate evexomostat and the pharmacologically active small molecule SDX-7539 in this patient population and document evidence of antitumor or antimetastatic activity. Exploratory outcomes were to evaluate the effects of evexomostat on key angiogenic biomarkers (bFGF, VEGF), metabolic biomarkers (insulin, leptin, adiponectin, and lipids) and inflammatory markers analyzed from patient serum samples.

Safety assessments included evaluation of DLTs, AEs, vital signs, electrocardiogram recordings, physical examinations, and laboratory tests. Local tolerability was assessed predose, on each treatment day after dosing, on days immediately following dosing, and weekly. AEs, including laboratory abnormalities, were graded using the NCI Common Terminology Criteria for Adverse Events v4.03 from the day of the first study dose until 28 days after the last dose.

Blood samples were collected to enable PK bioanalyses at the following timepoints: cycles 1, 3, and 6: predose, 3, 6, 24, 48, and 96 hours after dosing, and days 8 (predose on the Q7D schedule), 15 (predose) and 22 (predose on the Q7D schedule); cycles 2, 4, 5, 7, and beyond: days 1 (predose), 8 (predose on the Q7D schedule), 15 (predose), and 22 (predose on the Q7D schedule). PK samples were collected on day 1 (predose, 3, 6, and 24 hours) for cycle 1 and then predose days 1 and 15 for even cycles and predose for odd cycles thereafter. Analysis of exploratory biomarkers was conducted using commercially available ELISA kits.

The levels of the released small-molecule METAP2 inhibitor SDX-7539 in plasma were measured using a validated LC/MS-MS method with a lower limit of quantification (LLOQ) of 10.0 pg/mL. The levels of the polymer–drug conjugate evexomostat in plasma were measured using a fit-for-purpose LC/MS-MS method. Target engagement was assessed at various times postdose in lysed whole blood using a validated ELISA designed to measure uninhibited METAP2 (25).

All measurable lesions up to a maximum of 2 lesions per organ and 5 lesions in total, representative of all involved organs, were identified as target lesions, and all other lesions (or sites of disease) were identified as nontarget lesions. Tumor burden was scheduled to be performed with CT scans every 8 weeks and was evaluated according to RECIST v1.1. Assessments for target and nontarget lesions were conducted until disease progression was documented or patient withdrawal of consent.

The objective response rate was calculated as the percentage of patients with complete response (CR) or partial response (PR) at any time during the study. The disease control rate was calculated as the percentage of patients with CR, PR, or stable disease (SD) assessed in target lesions at any time during the study. Changes from baseline in nontarget lesions and the number and location of new metastatic lesions were recorded while the patient was on treatment.

### Statistical analysis

Analyses were conducted using SAS v 9.4 (SAS Institute) unless otherwise noted. AEs were coded in MedDRA v18.1 and analyzed by system organ class/preferred term. Treatment-related AEs (TRAE) were tabulated for all events occurring in two patients or more. Response was assessed using RECIST v1.1. PK analysis utilized WinNonlin v8.1 and a noncompartmental analysis approach.

### Data availability

Reports containing raw data as well as derived data supporting the findings of this study are available upon reasonable request from the corresponding author or through Vivli.org.

## Results

### Baseline characteristics

A total of 48 patients were screened for study eligibility and 32 were enrolled. The average number of prior lines of therapy was 4.1 (range was from 1–13; Table 1). Of the enrolled patients, the mean age was 68.0 years, and 56% were female. Seventy-eight percent of enrolled patients had an Eastern Cooperative Oncology Group status of 1. The primary diagnoses were for non–small cell lung cancer for eight (25%) patients, colon cancer for six patients (18.8%), breast cancer for four (13%) patients, rectal cancer for three (9%) patients, and hepatocellular or pancreatic cancer [each two patients (6%)]. All other primary diagnoses were single patients and are summarized in Table 1 and Supplementary Table S1, along with dose level and patient cohorts. All 32 patients also had received prior radiotherapy.

**Table 1:** Patient demographics and baseline clinical characteristics

Characteristics	All patients *N* = 32, *N* (%)
Age, median (range), years	68.0 (49–79)
Sex, *n* (%)	
Female	18 (56.3)
Male	14 (43.8)
Race, *n* (%)	
White	26 (81.3)
Asian	3 (9.4)
Black	2 (6.3)
Other	1 (3.1)
Obesity (BMI > 30), *n* (%)	
Female	9 (50)
Male	7 (50)
Screening Eastern Cooperative Oncology Group, *n* (%)	
0	7 (21.9)
1	25 (78.1)
Lines of prior systemic therapy, median (range)	4.1 (1–13)
Lines of prior radiotherapy, median (range)	1 (1–7)
Primary diagnosis, *n* (%)	
Non–small cell lung cancer	8 (25)
Colon cancer^a^	6 (18.8)
Breast cancer^a^	4 (12.5)
Rectal cancer^a^	3 (9.4)
Hepatocellular cancer^a^	2 (6.2)
Pancreatic cancer^a^	2 (6.2)
Bladder cancer	1 (3.1)
Cervical cancer	1 (3.1)
Appendiceal cancer	1 (3.1)
Carcinoid cancer	1 (3.1)
Endometrial cancer^a^	1 (3.1)
Ovarian cancer^a^	1 (3.1)
Small-cell lung cancer	1 (3.1)

a Cancers sensitive to obesity/metabolic dysfunction.

### Safety

Most TRAEs were of grade 1 or 2 in severity, nonserious, and did not lead to study drug discontinuation (Table 2). No deaths attributable to evexomostat were reported. The most common treatment-emergent AEs, regardless of dose, were fatigue (44%), decreased appetite (38%), constipation and nausea (each 28%), and diarrhea (22%; Table 3). All other TEAEs occurred at an incidence <20%.

**Table 2:** TRAEs occurring in >1 patient

	Number of patients (*N* = 32) *n* (%)	
Common TRAEs	Grade 1/2	Grade 3/4
Thrombocytopenia	10 (31)	4 (13)
Anorexia	12 (38)	1 (3)
Fatigue	11 (34)	1 (3)
Anemia	7 (22)	1 (3)
Electrocardiogram QTc interval prolonged	1 (3)	1 (3)
Vasculitis	1 (3)	1 (3)
Injection site reaction	8 (25)	0
Diarrhea	7 (22)	0
Nausea	5 (16)	0
Alopecia	4 (13)	0
Vomiting	4 (13)	0
Abdominal pain	3 (9)	0
Alkaline phosphatase increased	3 (9)	0
Dysgeusia	3 (9)	0
Hypoalbuminemia	3 (9)	0
Constipation	2 (6)	0
Creatine phosphokinase increased	2 (6)	0
Hyperglycemia	2 (6)	0
Hypocalcemia	2 (6)	0

**Table 3:** Common (overall incidence ≥10%) treatment-emergent AEs

MEDRA Terms	SDX-7320 dose (mg/m^2^)					
	1.7–36.0 Q7D	49.0 Q7D	36.0 Q14D	49.0 Q14D	65.0 Q14D	All patients
	(*N* = 10)	(*N* = 5)	(*N* = 6)	(*N* = 6)	(*N* = 5)	(*N* = 32)
	*N* (%)	*N* (%)	*N* (%)	*N* (%)	*N* (%)	*N* (%)
Any treatment-emergent AE	10 (100.0)	4 (80.0)	6 (100.0)	6 (100.0)	5 (100.0)	31 (96.9)
Fatigue	4 (40.0)	3 (60.0)	3 (50.0)	2 (33.3)	2 (40.0)	14 (43.8)
Decreased appetite	4 (40.0)	2 (40.0)	3 (50.0)	2 (33.3)	1 (20.0)	12 (37.5)
Constipation	3 (30.0)	3 (60.0)	1 (16.7)	0	2 (40.0)	9 (28.1)
Nausea	3 (30.0)	4 (80.0)	1 (16.7)	1 (16.7)	0	9 (28.1)
Anemia	3 (30.0)	1 (20.0)	1 (16.7)	2 (33.3)	1 (20.0)	8 (25.0)
Diarrhea	1 (10.0)	3 (60.0)	0	1 (16.7)	2 (40.0)	7 (21.9)
Abdominal pain	3 (30.0)	0	2 (33.3)	1 (16.7)	0	6 (18.8)
Dyspnea	1 (10.0)	2 (40.0)	1 (16.7)	2 (33.3)	0	6 (18.8)
Abdominal pain	1 (10.0)	2 (40.0)	0	2 (33.3)	0	5 (15.6)
Upper						
Vomiting	1 (10.0)	1 (20.0)	1 (16.7)	2 (33.3)	0	5 (15.6)
Platelet count	1 (10.0)	1 (20.0)	1 (16.7)	0	1 (20.0)	4 (12.5)
Decreased						
Back pain	3 (30.0)	0	1 (16.7)	0	0	4 (12.5)
Dysgeusia	1 (10.0)	2 (40.0)	1 (16.7)	0	0	4 (12.5)
Alopecia	3 (30.0)	0	0	0	1 (20.0)	4 (12.5)

DLTs consisting of reversible thrombocytopenia (grades 3 and 4) were reported for two patients at 49 mg/m^2^ on the Q7D dosing schedule following repeat drug administration (a patient with nonfebrile grade 4 thrombocytopenia received an infusion of platelets). Accordingly, the new dosing schedule was initiated (i.e., Q14D) at starting at 36 mg/m^2^. A DLT of vasculitis was experienced by one patient in the 36 mg/m^2^ Q14D cohort during cycle 1 (on C1D26), prompting cohort expansion at this dose level, with no subsequent DLTs seen. The vasculitis (grade 3) was diagnosed as leukocytoclastic vasculitis, and the patient was treated with oral prednisone and topical glucocorticoid cream, leading to resolution of this AE. Reversible (grade 3) thrombocytopenia occurred at 65 mg/m^2^ on the Q14D schedule. Thus, 49 mg/m^2^ Q14D was determined to be the MTD on the Q14D schedule.

The mean number of treatment cycles administered was 3.1, with a maximum number of 9.0.

### Disposition of patients

Of the 32 patients enrolled, 28 had a tumor assessment (88%). Fourteen patients discontinued evexomostat before reaching the nominal end of cycle 2. Reasons for discontinuation included progressive disease (PD; six patients; 19%), physician or patient decision (seven patients; 22%), and AE (one patient; 3%).

### PK and PD

**Figure 1:**
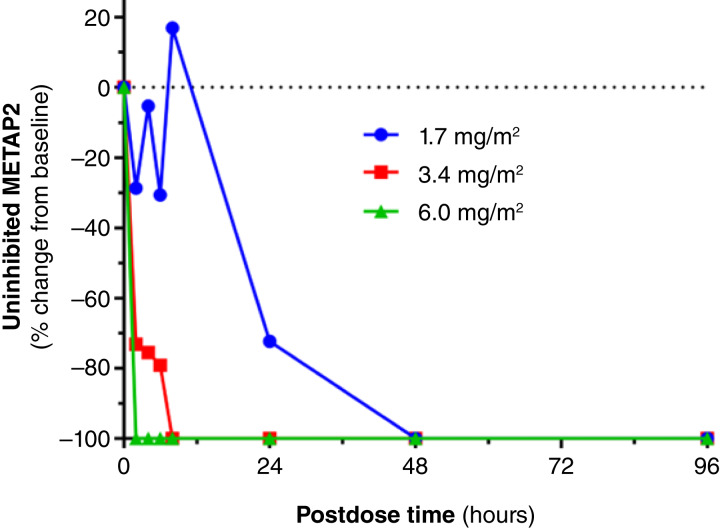
METAP2 inhibition measured in whole blood following increasing subcutaneous doses of SDX-7320. Blood samples obtained at the indicated times postdose were processed for measurement of uninhibited METAP2 using a capture ELISA as described in the “see Materials and Methods” section.

Inhibition of METAP2 measured in lysed whole blood in the first dose cohort reached 100% 48 hours postdose (1.7 mg/m^2^). Increasing dose levels were associated with decreased time to achieving 100% METAP2 inhibition (Fig. 1). Given the very low plasma concentrations of the released small molecule SDX-7539 in the first three dose cohorts (below the LLOQ of 20 pg/mL), coupled with METAP2 inhibition at low doses, it seems that inhibition of METAP2 occurs secondary to cellular uptake of evexomostat and intracellular release of SDX-7539.

**Figure 2:**
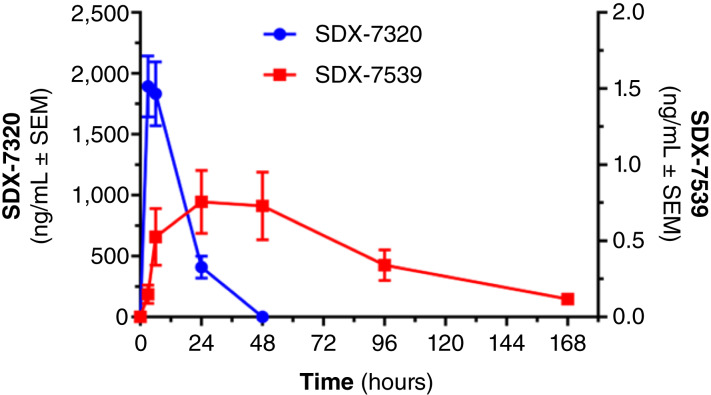
Exposure-time profile of SDX-7320 and SDX-7539 in cycle 1 (*n* = 5) in patients dosed at 49 mg/m^2^ SDX-7320. Plasma samples were prepared from blood drawn at the indicated times postdose. The levels of SDX-7320 and the small molecule SDX-7539 were measured by LC/MS-MS.

**Figure 3:**
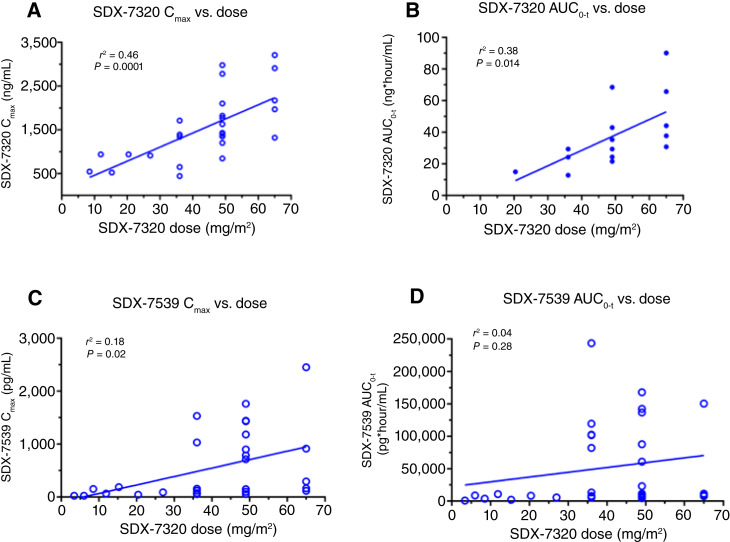
PK of polymer–drug conjugate SDX-7320 and released small molecule SDX-7539. PK parameters for SDX-7320 (C_max_ in **A** and AUC_0-t_ in **B**) and SDX-7539 (C_max_ in **C** and AUC_0-t_ in **D**) were determined from individual graphs of drug exposure vs. time and then plotted vs. the administered dose of SDX-7320.

Exposure of the polymer–drug conjugate evexomostat in plasma after subcutaneous dosing was rapid (T_max_ occurred between 3 and 6 hours; Fig. 2) and exhibited proportionality with increasing dose (Fig. 3A and B). The half-life of evexomostat could not be calculated because of limited availability of data after C_max_. In contrast, T_max_ of the released active moiety SDX-7539 was 29 hours (range, 6–96 hours; Fig. 2) and exhibited variability with increasing dose (Fig. 3C and D). Levels of SDX-7539 were sustained over several days, with detectable levels observed 7 days following a single dose. Analysis of plasma SDX-7539 exposure along with the change in platelets identified a potential relationship between C_max_ and the AUC in cycle 1 versus subsequent reductions in platelets (as % change from baseline to C1D8 or C1D15, Supplementary Fig. S1A and S1B, respectively). This observation prompted the change in dosing schedule from Q7D to Q14D. No relationship was identified between plasma levels of the parent polymer prodrug evexomostat in cycle 1 for either C_max_ or the AUC and subsequent reductions in platelets (Supplementary Fig. S2A and S2B, respectively).

No significant trends were seen with regard to changes from baseline in clinical chemistry, vital signs, or ECG findings after exposure to evexomostat. Based on safety findings with regards to thrombocytopenia, the RP2D and schedule for future clinical studies of evexomostat is 49 mg/m^2^ on the Q14D schedule.

### Antitumor activity

Best responses of SD or better were seen across the range of evexomostat doses evaluated, with no clear dose relationship seen. Of the 32 patients enrolled, 28 patients had at least one tumor burden assessment performed (the efficacy population). Of these 28 patients, three patients had unscheduled tumor assessments prior to a cycle 2 assessment and therefore were not considered part of the evaluable subset of the efficacy population. Twenty of the evaluable patients (20/25 evaluable, 80%) had at least one SD determination before leaving the study or experiencing PD (Table 4). Of these 20 patients, the period of measurable SD averaged 87 days (Supplementary Fig. S3). There was no CR or PR in this study. Ninety-six percent (24 of 25) of evaluable patients had no new metastatic lesions in the first 2 months of treatment, whereas 100% (*n* = 11) of patients saw no new metastatic lesions following four or more cycles of treatment (Supplementary Table S3). Non-CR/non-PD responses in nontarget lesions were noted in 8 of 11 patients at the cycle 4 tumor assessment (Supplementary Table S3).

**Table 4:** Response rates in the evaluable subset of the efficacy population

	1.7–49 mg/m^2^ Q7D (*N* = 14)	36–65 mg/m^2^ Q14D (*N* = 11)	All patients (*N* = 25)
Best overall response, *N* (%)			
CR	0	0	0
PR	0	0	0
SD	12 (85.7)	8 (72.7)	20 (80)
PD	2 (14.3)	3 (27.3)	5 (20)
NE	0	0	0
Objective response rate %, (*N*)			
	0 (0)	0 (0)	0 (0)
Disease control rate %, (*N*)			
	85.7 (12)	72.7 (8)	80 (20)

Abbreviations: NE, not evaluable.

### Biomarker assessments

**Figure 4:**
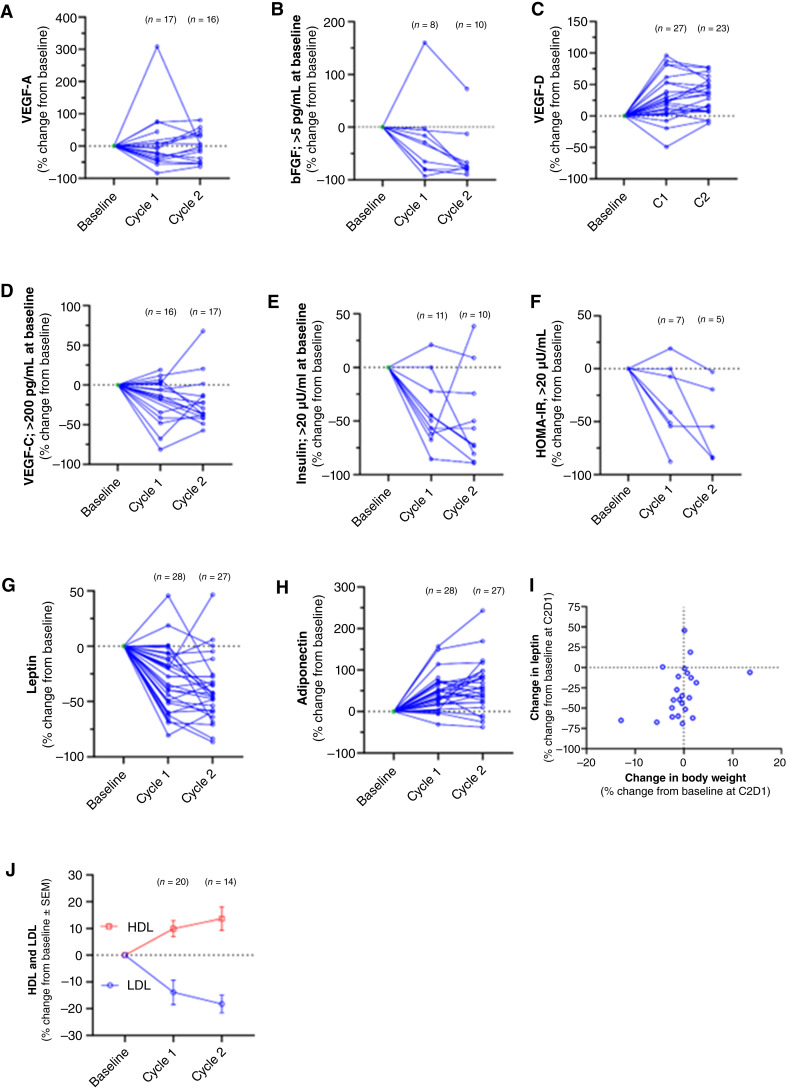
Changes in oncologic and metabolic biomarkers measured in cycles 1 and 2. Note that values < LLOQ were assumed to be LLOQ/2 for the purposes of graphical representation. Plasma levels of the biomarkers were measured by specific immunoassays at baseline (C1D1), in cycle 1 and cycle 2 and presented as the percent change from baseline for VEGF-A (**A**), bFGF, stratified for baseline levels >5 pg/mL (**B**), VEGF-D (**C**), VEGF-C, stratified for baseline levels >200 pg/mL (**D**), insulin, stratified for baseline levels >20 μU/mL (**E**), HOMA-IR, from patients with baseline insulin >20 μU/mL (**F**), leptin (**G**), adiponectin (**H**), change in leptin vs. change in body weight in cycle 1 (**I**), and change in HDL and LDL (**J**).

Overall, 32 patients contributed biomarker sample data. Consistent improvements in response to evexomostat were observed with respect to the angiogenic biomarkers VEGF-C and bFGF (FGF2) for patients with elevated baseline levels of these proteins (Fig. 4A and B). Plasma levels of VEGF-A exhibited a mixed response whereas plasma levels of VEGF-D increased in most patients (Fig. 4C and D).

Consistent with known effects of METAP2 inhibition, patients with baseline insulin resistance (fasting insulin >20 mU/mL; *n* = 11) exhibited decreased fasting insulin after treatment with evexomostat, and a subset of those patients (*n* = 7) for whom time-matched fasting insulin and fasting glucose were available showed marked improvements in their insulin resistance, based on a decline in the homeostatic model assessment of insulin resistance (HOMA-IR) score (Fig. 4E and F).

Decreases in the adipokine leptin were observed in most patients (27 of 31; Fig. 4G), whereas levels of the adipose tissue–derived hormone adiponectin increased in nearly all patients (28 of 31; Fig. 4H). Surprisingly, no relationship was observed between change in body weight and change in leptin from baseline to the end of cycle 1 (Fig. 4I), suggesting that the initial metabolic PD effects of evexomostat are independent of weight loss and may reflect direct effects of METAP2 inhibition within adipose tissue.

In addition to improvements in key metabolic hormones, favorable and significant changes in patient blood lipid profiles were noted, specifically increases in HDL and matching decreases in LDL (Fig. 4J).

## Discussion

This phase I dose-escalation study investigated the safety and tolerability of evexomostat (SDX-7320), an optimized METAP2 inhibitor, in 32 patients with heavily pretreated, late-stage cancer with a variety of solid tumors and metabolic states. The results from the study indicate that evexomostat was well-tolerated, with reversible thrombocytopenia being the DLT observed at 49 mg/m^2^ on the Q7D dosing schedule and at 65 mg/m^2^ on the Q14D schedule, contemporaneous with increasing exposure to the released, active small molecule SDX-7539 following repeat administration. Thrombocytopenia is anticipated to be a drug class effect, as it has also been observed in a recent clinical trial of a non–fumagillin-based, reversible METAP2 inhibitor (1). A DLT of leukocytic vasculitis was observed in one patient at 36 mg/m^2^ and was listed as possibly related to the study drug. However, dose expansion at 36 mg/m^2^ was uneventful, and no additional vasculitis AEs were observed.

Evexomostat was well-tolerated, with most TRAEs being of grade 1 or 2 in severity, nonserious, and generally did not lead to study drug discontinuation. In prior clinical trials of fumagillin-based small-molecule METAP2 inhibitors (e.g., TNP-470), dose-limiting CNS toxicities were observed (17, 18), preventing further clinical development. In contrast, evexomostat exhibited no dose-limiting CNS toxicities, likely due to the benefits of drug conjugation and low systemic exposure of the released small molecule, although measurement of drug exposure in cerebrospinal fluid was not specifically conducted in these patients.

METAP2 inhibitors have previously been clinically shown to have antitumor and antimetastatic effects in patients with late-stage cancer, as well as resensitizing patients to chemotherapy when combined with such agents (3, 17, 18, 26, 27). In this study, SD was achieved in a majority of patients (20 of 25 evaluable), all of whom had disease progression prior to entering the study. Ninety-six percent of evaluable patients had no new metastases in the first 2 months of treatment, whereas 100% of patients had complete metastatic control following four or more cycles of treatment (Supplementary Table S2), affirming the antimetastatic activity of this drug class (28).

Fumagillin-class molecules have been reported to possess potent antiangiogenic properties (29, 30) and improve systemic metabolic status (31, 32). The effects of evexomostat in this phase 1 safety study on key angiogenic (bFGF and VEGF-C) and metabolic biomarkers (insulin, leptin, adiponectin, and lipids) suggested potent antiangiogenic and metabolic hormone regulation. In contrast to decreased VEGF-C, plasma levels of VEGF-D increased in many patients treated with evexomostat. Treatment with the small-molecule VEGF receptor tyrosine kinase inhibitor sunitinib was previously shown to increase circulating VEGF-D, which may reflect a compensatory response to inhibition of VEGF receptor or downstream pathways (33, 34). Despite favorable changes in metabolic biomarkers in response to evexomostat in this study, no clear relationship between these changes and cancer-associated clinical parameters (i.e., change in tumor burden, time-to-last tumor assessment) were observed. There was, however, a trend to increased time-to-last-tumor assessment with increased adiponectin measured in the first cycle of treatment (Supplementary Fig. S4). Many factors likely constrained our ability to establish relationships between changes in biomarkers and cancer-related measures, including the multiple dose levels of evexomostat (11 different dose levels, eight of which were single-patient cohorts), heterogenous cancer types, the late stage of patients’ disease, and multiple prior lines of treatment.

As this clinical trial was designed as a phase I safety study, there were no inclusion criteria around obesity or metabolic dysfunction. However, 50% of the patients enrolled in this study were obese [i.e., body mass index (BMI) > 30 kg/m^2^] and 10 patients (31%) had baseline insulin resistance, but there was limited overlap between the two populations: of the patients with insulin resistance at baseline, only three were obese, underscoring the limitations of BMI as a predictor of metabolic dysfunction. Furthermore, decreases in plasma leptin in cycle 1 were not associated with any change in body weight or BMI, suggesting that evexomostat has direct effects on adipose tissue. In this trial, the effects of evexomostat on markers of metabolic dysfunction seem to be independent of weight loss, in contrast to other small-molecule METAP2 inhibitors such as CKD-732/beloranib. We did, however, observe fluctuations in body weight and body weight change from baseline over time for the patients enrolled in this trial, as shown in Supplementary Fig. S5A and S5B, respectively.

Evexomostat is an optimized METAP2 inhibitor with improved pharmacologic properties, a convenient 2-week (Q14D), subcutaneous dosing regimen, and no significant CNS toxicity reported to date. Its favorable safety profile, together with possible antimetastatic activity as well as antiangiogenic and antimetabolic PD activities, make it an ideal candidate for combination treatment with standard-of-care therapies in the broad spectrum of cancers sensitive to dysregulated metabolic hormones. Evexomostat is a first-in-class agent in metabo-oncology, an emerging field in cancer research focused on the intersection of obesity/metabolic dysfunction and cancer outcomes.

Currently, evexomostat is being investigated in two phase 1b/2 clinical proof-of-concept studies: in second-line metastatic HR+/Her2− breast cancer in combination with either the PI3Kα inhibitor alpelisib or the AKT inhibitor capivasertib plus fulvestrant (the Amelia-1 study – www.amelia1.com) and, in collaboration with Memorial Sloan Kettering Cancer Center, in first- to third-line metastatic triple-negative breast cancer in combination with eribulin in women with baseline metabolic dysfunction (the Aretha study – www.aretha1.com).

## Supplementary Material

Figure S1 Relationship between plasma SDX-7539 Cmax or AUC and change in platelets

Figure S2 Relationship between plasma SDX-7320 Cmax or AUC and change in platelets

Figure S3 Time to last tumor assessment

Figure S4 Time to last tumor assessment versus plasma adiponectin

Figure S5 Body weight and body weight change

Table S1 Representativeness of the study participants

Table S2 Patient demographics

Table S3 Target lesion and non-target lesion responses
