# Supplementary figures and images for: A Phase 1 Safety Study of Evexomostat (SDX-7320) in Patients with Late-Stage Cancer: An Antiangiogenic, Insulin-Sensitizing Drug Conjugate Targeting METAP2
Source: Cancer Res Commun. 2025 Jun 23;5(6):1008–17. doi: 10.1158/2767-9764.CRC-24-0627 (PMC12183619; doi:10.1158/2767-9764.CRC-24-0627)

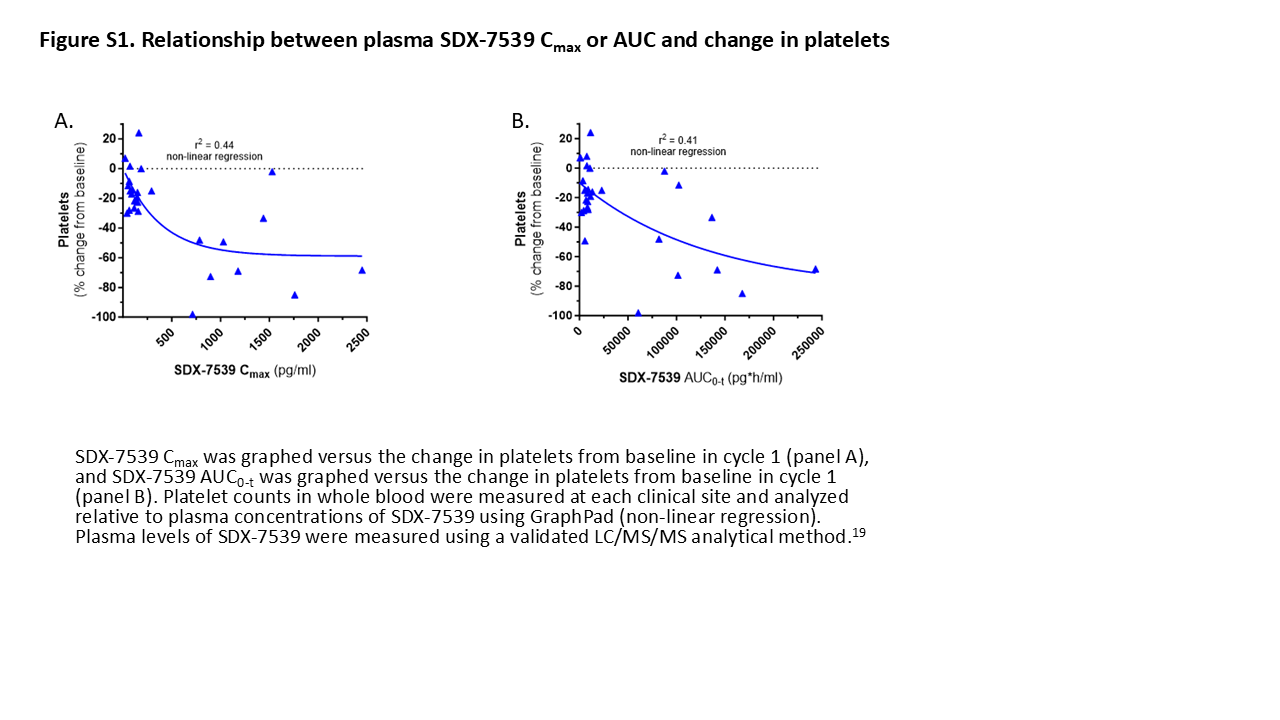

Supplement: Figure S1 — Relationship between plasma SDX-7539 Cmax or AUC and change in platelets [file crc-24-0627_figure_s1_suppsf1.png]

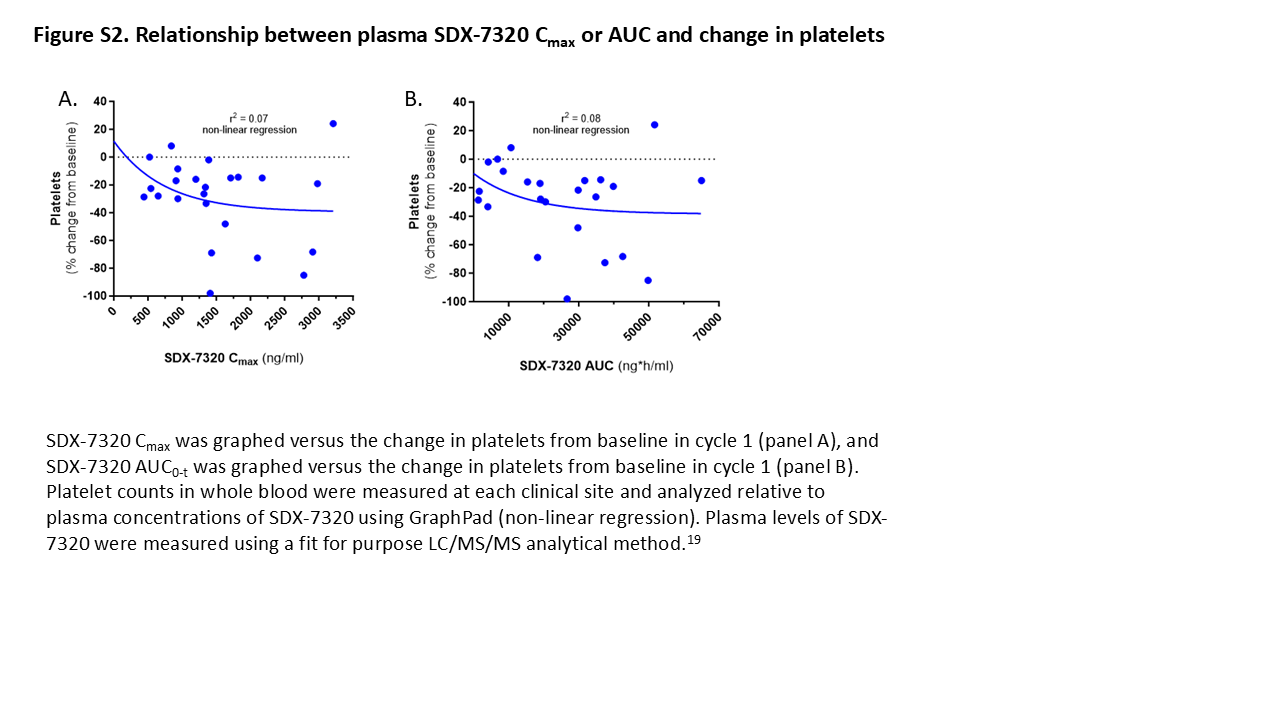

Supplement: Figure S2 — Relationship between plasma SDX-7320 Cmax or AUC and change in platelets [file crc-24-0627_figure_s2_suppsf2.png]

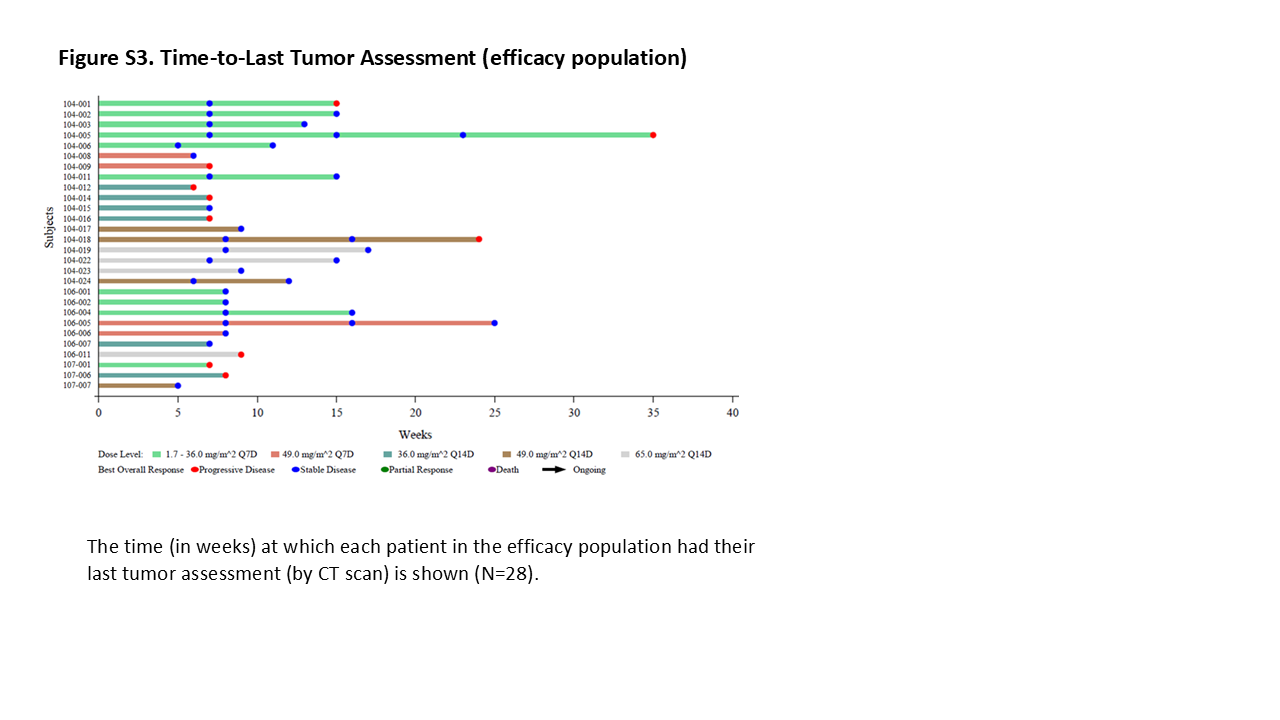

Supplement: Figure S3 — Time to last tumor assessment [file crc-24-0627_figure_s3_suppsf3.png]

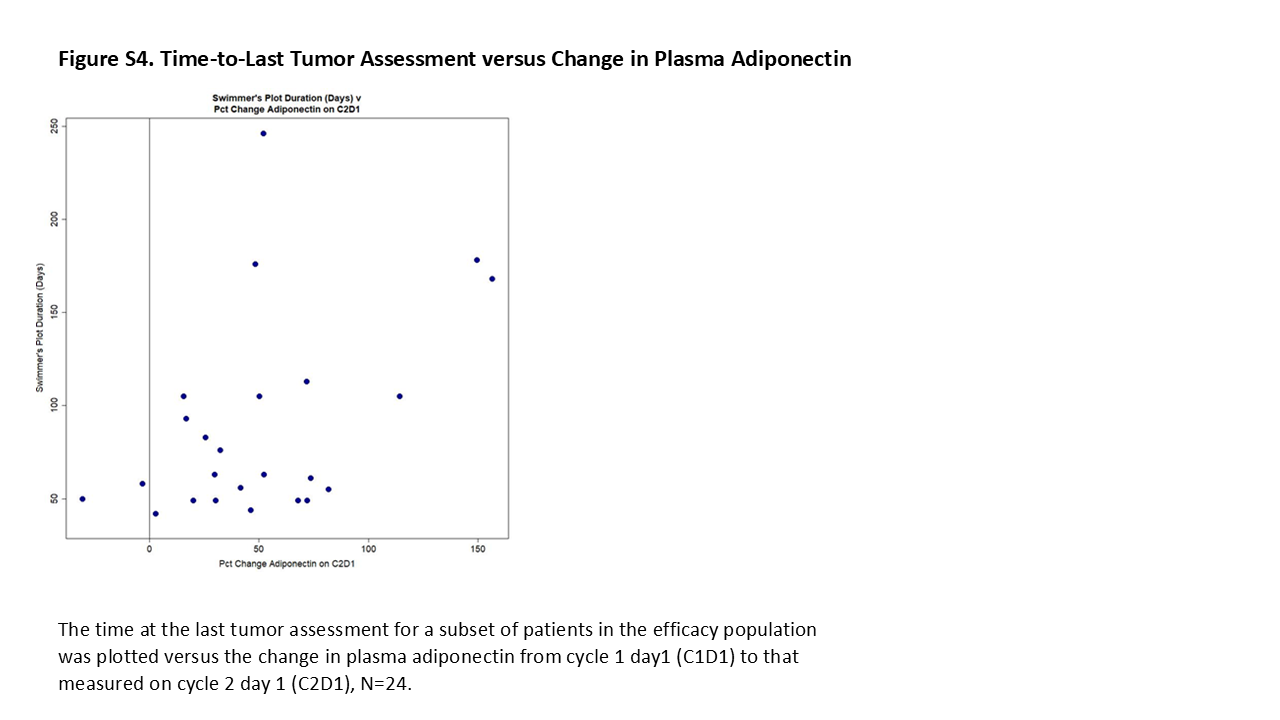

Supplement: Figure S4 — Time to last tumor assessment versus plasma adiponectin [file crc-24-0627_figure_s4_suppsf4.png]

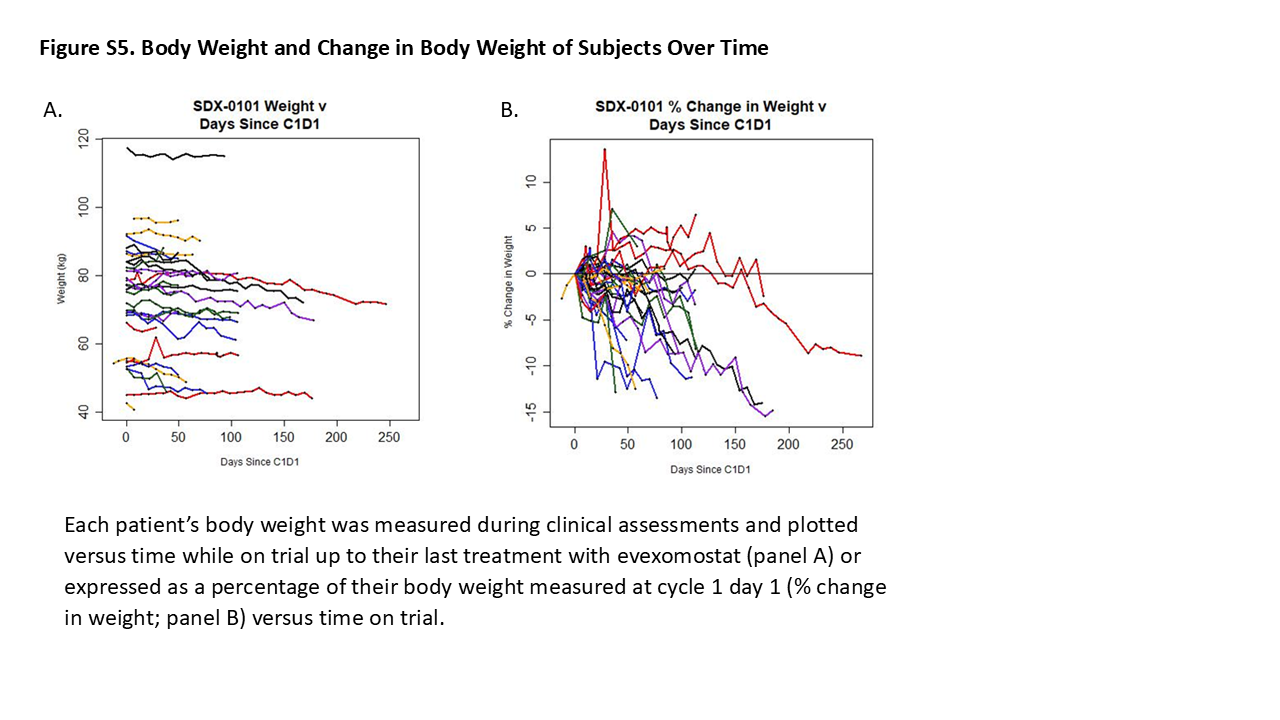

Supplement: Figure S5 — Body weight and body weight change [file crc-24-0627_figure_s5_suppsf5.png]

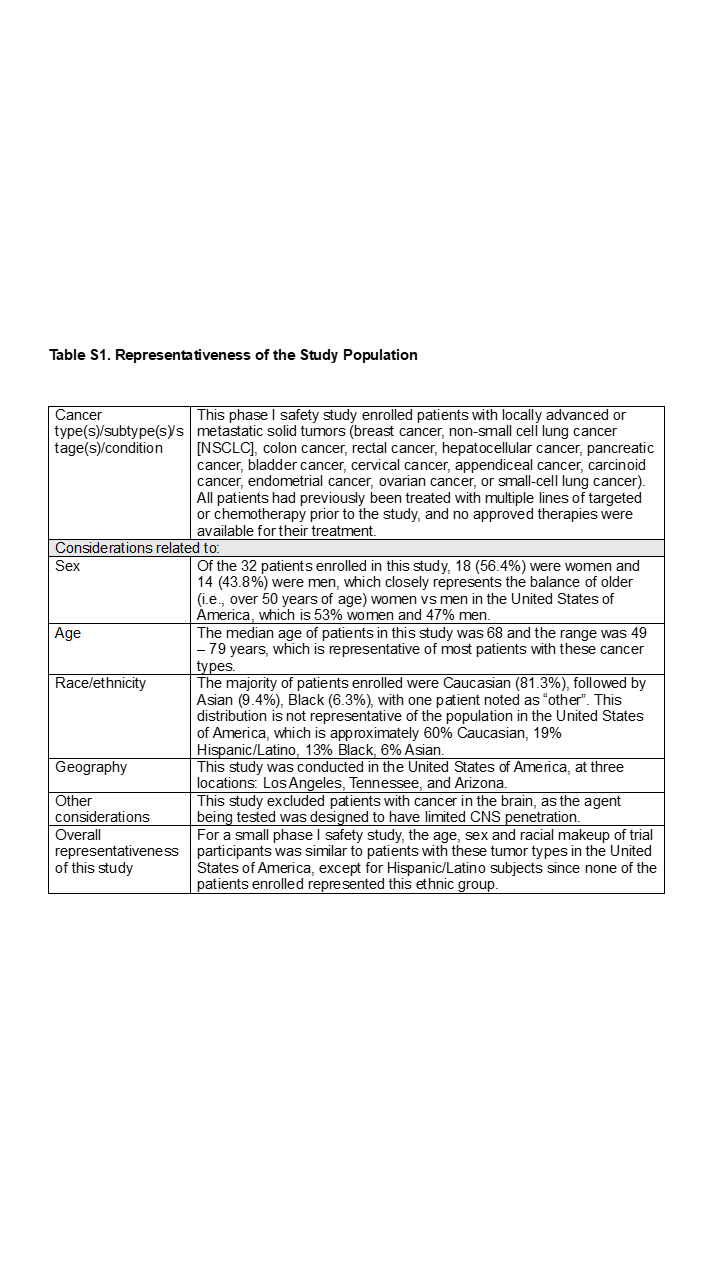

Supplement: Table S1 — Representativeness of the study participants [file crc-24-0627_table_s1_suppst1.png]

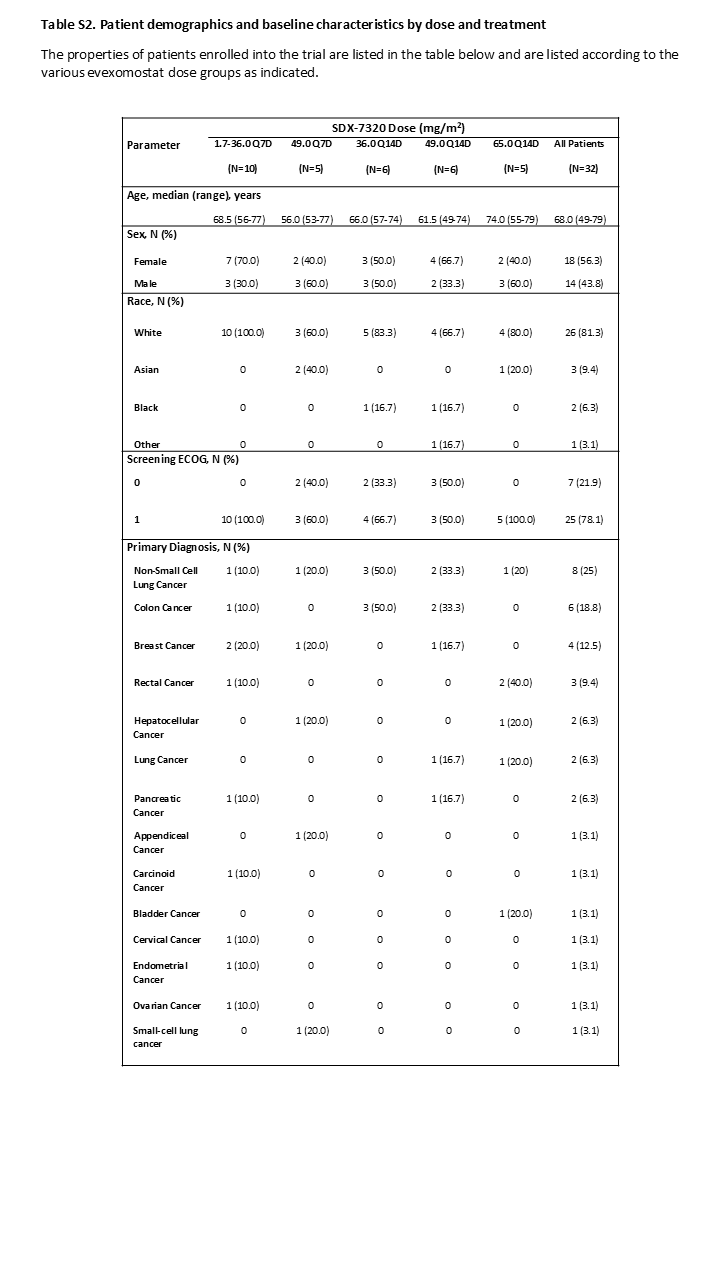

Supplement: Table S2 — Patient demographics [file crc-24-0627_table_s2_suppst2.png]

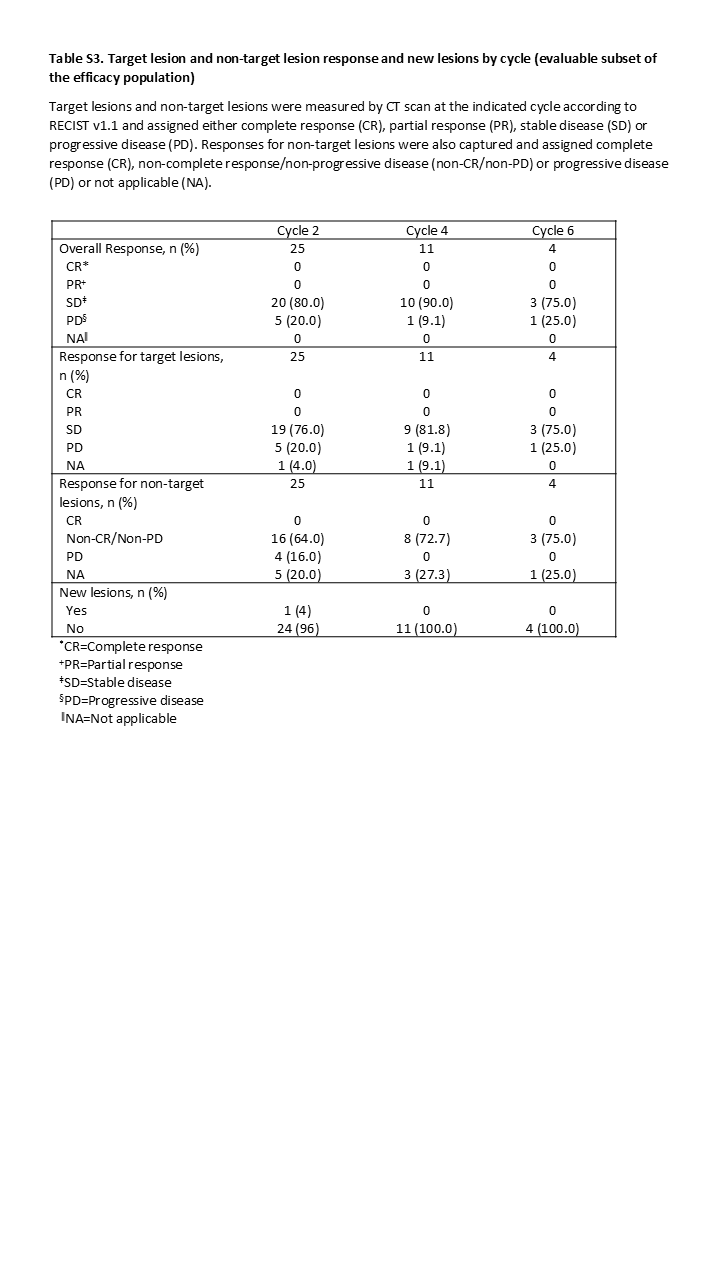

Supplement: Table S3 — Target lesion and non-target lesion responses [file crc-24-0627_table_s3_suppst3.png]
